# *Lactiplantibacillus plantarum* as a Potential Adjuvant and Delivery System for the Development of SARS-CoV-2 Oral Vaccines

**DOI:** 10.3390/microorganisms9040683

**Published:** 2021-03-26

**Authors:** Julio Villena, Chang Li, Maria Guadalupe Vizoso-Pinto, Jacinto Sacur, Linzhu Ren, Haruki Kitazawa

**Affiliations:** 1 Reference Centre for Lactobacilli (CERELA-CONICET), Laboratory of Immunobiotechnology, Tucuman CP4000, Argentina; 2 Laboratory of Animal Products Chemistry, Food and Feed Immunology Group, Graduate School of Agricultural Science, Tohoku University, Sendai 980-8572, Japan; 3 Research Unit of Key Technologies for Prevention and Control of Virus Zoonoses, Chinese Academy of Medical Sciences, Military Veterinary Institute, Academy of Military Medical Sciences, Changchun 130122, China; lichang78@163.com; 4 Infection Biology Laboratory, Instituto Superior de Investigaciones Biológicas (INSIBIO), CONICET-UNT, Tucuman CP4000, Argentina; mgvizoso@fm.unt.edu.ar (M.G.V.-P.); jacintosacur@gmail.com (J.S.); 5 College of Animal Sciences, Key Lab for Zoonoses Research, Ministry of Education, Jilin University, Changchun 130062, China; 6 International Education and Research Center for Food Agricultural Immunology, Livestock Immunology Unit, Graduate School of Agricultural Science, Tohoku University, Sendai 980-8572, Japan

**Keywords:** *Lactiplantibacillus plantarum*, COVID-19, SARS-CoV-2, vaccine, immunobiotics, recombinant lactobacilli, mucosal immunity, antiviral immunity

## Abstract

The most important characteristics regarding the mucosal infection and immune responses against the Acute Respiratory Syndrome coronavirus 2 (SARS-CoV-2) as well as the current vaccines against coronavirus disease 2019 (COVID-19) in development or use are revised to emphasize the opportunity for lactic acid bacteria (LAB)-based vaccines to offer a valid alternative in the fight against this disease. In addition, this article revises the knowledge on: (a) the cellular and molecular mechanisms involved in the improvement of mucosal antiviral defenses by beneficial *Lactiplantibacillus plantarum* strains, (b) the systems for the expression of heterologous proteins in *L. plantarum* and (c) the successful expressions of viral antigens in *L. plantarum* that were capable of inducing protective immune responses in the gut and the respiratory tract after their oral administration. The ability of *L. plantarum* to express viral antigens, including the spike protein of SARS-CoV-2 and its capacity to differentially modulate the innate and adaptive immune responses in both the intestinal and respiratory mucosa after its oral administration, indicates the potential of this LAB to be used in the development of a mucosal COVID-19 vaccine.

## 1. Introduction

Coronaviruses are positive-sense single-stranded RNA (ssRNA) viruses with a wide range of hosts. To date, seven human coronaviruses (HCoV) were identified as human-pathogens; HCoV-229E, HCoV-OC43, HCoV-NL63 and HCoV-HKU1 (responsible for non-sever common cold), the Severe Acute Respiratory Syndrome coronavirus (SARS-CoV) isolated in 2003 in China and the Middle East Respiratory Syndrome coronavirus (MERS-CoV) that emerged in Middle Eastern countries in 2012 [1]. Both SARS-CoV and MERS-CoV are highly pathogenic viruses that caused nosocomial outbreaks with high case-fatality rates. The seventh and most recently identified human coronavirus is the SARS-CoV-2, responsible for the coronavirus disease 2019 (COVID-19).

COVID-19 emerged in December 2019 in Wuhan, China, and rapidly spread worldwide in a few months due to its high transmissibility and pathogenicity. Although SARS-CoV-2 induce a milder clinical commitment than SARS-CoV or and MERS-CoV, COVID-19 has affected more than 100 million people worldwide, causing the death of 2,217,005 persons according to the WHO’s situation report on 1 February 2021 (WHO, 2021) [2].

Several transmission-mitigation strategies have been implemented in most countries, including social distancing and lockdowns. In addition, a vaccine development race started as never seen before. Currently, several COVID-19 vaccines have finished the phase III clinical testing or been granted an emergency use authorization, including BBIBP-CorV (Sinopharm) and CoronaVac (Sinovac) in China, Pfizer-BioNTech COVID-19 vaccine (Pfizer) and mRNA-1273 vaccine (Moderna) in the United States, and Sputnik-V vaccine in Russia, offering hope for controlling the SARS-CoV-2 infection and stop the pandemic in the near future [3,4,5].

Since the protective immune responses against SARS-CoV-2 are poorly understood, it is unclear which vaccine strategies will be the most successful. The majority of COVID-19 vaccines have been designed to induce anti-SARS-CoV-2 neutralizing antibodies to prevent virus entry into the target cells. In some cases, vaccines are designed to induce both humoral and cellular immunity that could help limiting viral replication in the infected host [5]. Of note, most of the vaccines are designed for parenteral use, and therefore, are capable of mainly inducing systemic immunity despite of the fact that SARS-CoV-2 infects mucosal tissues and that human-to-human transmission is mediated by respiratory droplets and the fecal-oral transmission has not been ruled out [6,7]. The need to generate not only humoral but also cellular immunity against SARS-CoV-2 and to induce protective immunity in the mucosal surfaces where this virus initiates its replication allows us to speculate that this first generation of COVID-19 vaccines should be replaced later by a new generation of vaccines that allow overcoming the aforementioned limitations.

Protective mucosal immune responses are most effectively induced by mucosal immunization through oral or nasal routes, whereas injected vaccines are generally poor inducers of mucosal immunity. However, the induction of mucosal immune responses is challenging due to the physical-chemical barriers of the mucosal surfaces and the tendency to induce tolerance [8]. Therefore, mucosal vaccine delivery systems require high doses of antigens and efficient mucosal adjuvants. Lactic acid bacteria (LAB) have been proposed as both delivery vectors and mucosal adjuvants [9,10]. In the last decades, recombinant LAB have been tested as new-generation oral vaccine vectors due to their natural resistance to gastrointestinal conditions and their ability to modulate both intestinal innate and adaptive immune responses. In this sense, *Lactiplantibacillus plantarum* (Basonym *Lactobacillus plantarum*) is a good candidate for developing oral vaccines because it survives gastrointestinal conditions transiently colonizing the intestinal tract, it beneficially modulates the mucosal immune responses not only locally (intestinal mucosa) but in distant mucosal sites as well (respiratory mucosa) and there are molecular techniques available for the manipulation of its genome.

Here, we revise relevant information about SARS-CoV-2 infection at mucosal sites, the immune response against the virus and the current COVID-19 vaccines in development, and analyze the possibility of LAB-based vaccines to offer an alternative in the fight against this disease. In addition, we review: (a) the cellular and molecular mechanisms involved in improving mucosal antiviral defenses by *L. plantarum* strains, (b) the heterologous proteins expression systems for *L. plantarum* and (c) the protective immune responses in the gut and the respiratory tract after the oral administration of recombinant *L. plantarum*.

## 2. SARS-CoV-2 Mucosal Infections

**Figure 1:**
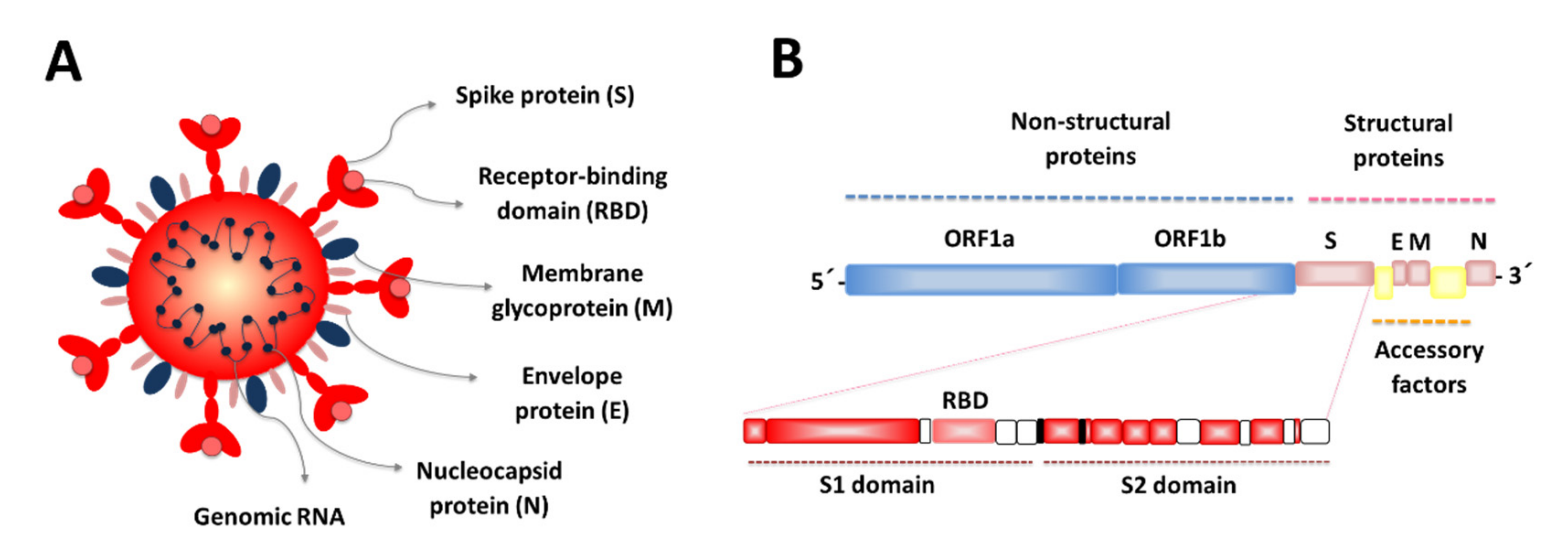
SARS-CoV-2 virion (**A**) and genome (**B**) structures. The virion contains a nucleocapsid composed of genomic RNA and N protein, which is enclosed inside the virus envelope consisting of S, E and M proteins. Approximately two-thirds of the RNA genome encodes a large polyprotein (ORF1a/b), while the last third proximal to the 3′-end encodes four structural proteins: spike (S), envelope (E), membrane (M) and nucleocapsid (N). The structure of the S-protein showing the S1 and S2 domains and the receptor-binding domain (RBD) are highlighted.

The viral 29.9 kb genome of SARS-CoV-2 encodes four structural proteins designated as spike (S), envelope (E), membrane (M) and nucleocapsid (N) proteins, respectively. The genome also encodes non-structural proteins and accessory factors (Figure 1).

**Figure 2:**
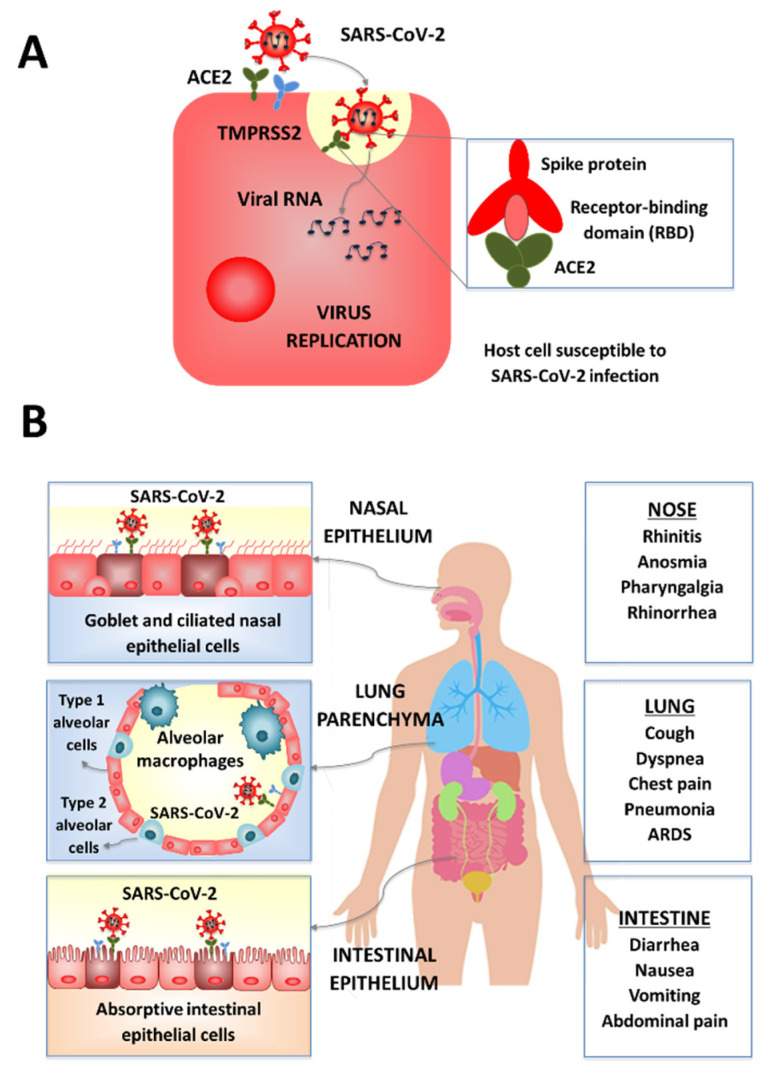
Mucosal tissues susceptible to SARS-CoV-2 infection. The virus infects mucosal cells expressing the surface receptors ACE2 and TMPRSS2 (**A**), and the active replication cause the death of cells, inducing respiratory and intestinal alterations (**B**).

Functional and genomic studies comparing SARS-CoV and SARS-CoV-2 showed that the spike protein (or S protein) of SARS-CoV-2 acts as the entry receptor for this virus. SARS-CoV-2 receptor recognition and attachment are initiated via interactions between the receptor-binding domain (RBD) of the S protein and the human angiotensin converting enzyme 2 (ACE2) expressed in numerous cells (Figure 2). Additionally, SARS-CoV-2 entry requires the efficient priming of the S protein by serine proteases such as the TMPRSS2 enzyme [11,12]. Hence, cell populations in human tissues and organs with higher ACE2 and TMPRSS2 expression, such as the nasal and bronchial epithelium and the alveoli, are more vulnerable to SARS-CoV-2 infection. Those cells include epithelial cells of the airways, the lung parenchyma, the small intestine (Figure 2) as well as the cells in vascular endothelia, and renal, liver and cardiovascular tissues [12,13], which explains the great diversity of symptoms that COVID-19 patients develop. These symptoms vary from patient to patient; however, most symptomatic patients have signs of affection of the airways and the gastrointestinal tract. The efficiency with which the SARS-CoV-2 binds to ACE2 in mucosal tissues was proposed as a key determinant of transmissibility, as shown by the higher binding affinity of SARS-CoV-2 to ACE2 than SARS-CoV [12].

### 2.1. SARS-CoV-2 Respiratory Infection

The clinical manifestations of COVID-19 range from mild respiratory symptoms to sever acute respiratory distress syndrome (ARDS); the most common symptoms are dry cough, rhinitis, sore throat, breath shortness, chest pain, myalgia, fatigue and pneumonia [14,15,16,17]. In the severe cases often needing hospitalization, complications such as ARDS, secondary infections and cardiac alterations appear [14].

Nasal epithelial cells belong to the cell populations with the highest expression of ACE2 in the respiratory tree [18,19]. ACE2 is expressed in goblet and ciliated cells of the nasal epithelium, as corroborated by in vitro scRNA-seq studies in primary air-liquid interface nasal cell cultures. The viral replication causes cytopathic effects and stops cilia movements [20,21]. The nasal epithelium also contains olfactory sensory neurons and accessory cells (ciliated, goblet and secretory cells as well as reserve basal stem cells, which express ACE2 [22]) responsible for detecting odors. The SARS-CoV-2 infection of the support cells in the olfactory epithelia together with the local inflammation cause the anosmia often seen in COVID-19 patients [22,23].

As mentioned earlier, ACE2 and TMPRSS2 are also highly expressed in type II pneumocytes (Figure 2), allowing SARS-CoV-2 replication in the lower respiratory tract [20]. The viral lyses of type II pneumocytes impairs their function of keeping the alveolar structure, which in turn reduces the gas exchange function of the lung [21]. Lung injuries are characterized by diffuse reactive hyperplasia of type II pneumocytes, diffuse alveolar damage, edema and the presence of proteinaceous or fibrin alveolar exudates [14]. In more severe cases of lower respiratory infection, interstitial fibroblasts proliferate thickening the alveolar septa and a hyaline membrane is formed. In addition, because of inflammation, a pronounced interstitial infiltration of mononuclear cells can be observed. These infiltrations can be accompanied by deposition of neutrophils in the intra-alveolar space, especially when a secondary bacterial infection is present [14]. Of note, endothelial cells expressing high ACE2 levels in the lung vasculature are also susceptible to SARS-CoV-2 infection, and their damage alters the alveolar–capillary barrier [14,19,20].

### 2.2. SARS-CoV-2 Intestinal Infection

Variable levels of ACE2 and TMPRSS2 have been detected in different cell types [24,25,26,27,28,29,30,31,32] including the epithelial subtype cells of the gastrointestinal tract (Figure 2) [26,29,31,32]. The highest ACE2 expression was reported for absorptive intestinal epithelial cells of the small intestine, especially in the ileum and the jejunum [20,33,34]. Further evidence of SARS-CoV-2 replication in the intestinal mucosa were the detection of nucleocapsid protein detection in duodenal epithelial cells [35], and of SARS-CoV-2 RNA in stool [33,36,37] and rectal [7,38] samples of COVID-19 patients [7,38]. The main gastrointestinal symptoms observed in COVID-19 adult patients were nausea or vomiting (1–10%), diarrhea (2–10%) [39,40] and abdominal pain (2–6%) [41,42]. In a study following COVID-19 pediatric patients, diarrhea was the main sign, reported in 3 out of the 10 infected children [7].

The clinical and experimental evidence indicate that SARS-CoV-2 can effectively infect and replicate in the intestinal mucosa, which has important implications for the disease management, patient care and infection control [40]. For instance, fecal viral shedding can be a source of infective aerosols generated from the toilet plume, leading to fomite transmission [43]. This transmission route could be particularly relevant considering that around 50% of patients tested positive for SARS-CoV-2 RNA in intestinal samples, and some remained positive for intestinal SARS-CoV-2 shedding after showing negative in their respiratory samples. Then, it has been suggested that viral shedding from the intestinal mucosa could be abundant and may last long after the resolution of respiratory symptoms [7,38].

## 3. SARS-CoV-2 Immune Response and Vaccines

### 3.1. SARS-CoV-2 Immune Response

The pathophysiology of COVID-19 resembles that of SARS-CoV infection, characterized by aggressive inflammatory responses that damage the infected tissues. Thus, the severity of COVID-19 depends not only on the SARS-CoV-2-induced cellular injury but also on the host response. The rapid viral replication induces extensive destruction of epithelial cells, the release of pro-inflammatory cytokines/chemokines and the recruitment of inflammatory cells into the infected tissues [44]. Therefore, both the ability of the host to control SARS-CoV-2 replication and to regulate the inflammatory response determine the outcome of COVID-19 [44,45,46]. The main aspects to be considered for understanding the host response to the virus are:

(i) Type I interferon-dependent immunity. During replication, pathogen-associated molecular patterns (PAMPs) are exposed, which are recognized by pattern recognition receptors (PRRs) expressed in both immune and non-immune cells. [47,48]. The PAMPs-PRRs interactions activate signaling pathways that induce type I and II interferons (IFNs) and inflammatory cytokines. IFNs, particularly IFN-β, promote an antiviral state through the up-regulation of hundreds of interferon-stimulated genes (ISGs) on neighboring immune and non-immune cells. The proper and timely production of type I IFNs in the mucosal tissues is, therefore, crucial to suppress viral replication and dissemination at an early stage. Coronaviruses such as SARS-CoV-2 are able to evade immune detection and dampen this initial type I IFNs-mediated antiviral response [44,45,46]. The failure in the early production of type I IFNs has been associated with the development of more severe COVID-19 cases [16,45,49]. Interestingly, a clinical and genome sequencing study evaluating patients with life threatening SARS-CoV-2 respiratory infection reported the presence of mutations in the key genes TLR3, IRF7 and IFNAR1 involved in the signaling pathways leading to the antiviral effect of type I IFNs [50]. Moreover, a clinical trial evaluating the levels of type I IFNs and the SARS-CoV-2 titers in blood samples from patients with severe or critical COVID-19 reported that a lower production of type I IFNs correlated with increased viral load in the blood [51]. Moreover, the same study described that the inefficient early production of type I IFNs was associated with an exacerbation of the inflammatory response. In line with these findings, reports have observed that reduced production of type I and type III IFN in patients with COVID-19 are accompanied by elevated secretion of pro-inflammatory chemokines and cytokines, which contribute to aggravate the COVID-19 pathology [49,52]. In severe cases of COVID-19, increased numbers of inflammatory monocytes and neutrophils in blood and CD14^+^CD16^+^ monocyte-derived macrophages in the respiratory tract were detected [3].

(ii) DC activation. The proper activation and regulation of the innate antiviral immunity mechanisms are necessary not only to control infection in the early stages, but also to induce adequate adaptive responses. Similar to SARS-CoV and MERS-CoV, SARS-CoV-2 suppresses DCs activation by dampening IFN signals [53]. Furthermore, the expression of HLA-DMA, HLA-DMB, HLA-DRB1 and CD74 is significantly diminished in severe cases of COVID-19, according to a transcriptomic study [54]. Consequently, SARS-CoV-2 impairs the adaptive immune responses by affecting antigen presentation.

(iii) T-cell-mediated immunity. Numerous studies have shown remarkable alterations in adaptive immunity in the most severe cases of COVID-19. Generally, independent of the severity of COVID-19 disease, CD8^+^ T cells seem to be more activated than CD4^+^ T cells [55]. Reduced numbers of both CD4^+^ and CD8^+^ T cells in blood samples are consistently observed in patients suffering COVID-19, particularly in more severe cases [7,25,56,57]. Moreover, T cell receptor sequencing demonstrated a greater TCR clonality of blood [58] and respiratory tract T cells [59] of COVID-19 patients suffering a mild disease compared to severe cases. Furthermore, the extent of blood CD8^+^ T cells reduction in intensive care patients correlates with COVID-19-associated disease mortality [55]. Some qualitative changes in the CD8^+^ T cell population were also described in severe COVID-19 cases, including the enhanced expression of exhaustion markers [60,61] and the diminished expression of CD107a and granzyme B [61,62]. On the other hand, CD4^+^ T cellular functionality is also impaired in critically ill COVID-19 patients: there is a significant reduction of IFN-γ producing CD4^+^ T cells [56,63,64]. In contrast, robust T cell responses specific for SARS-CoV-2 proteins N, M and S were detected by IFN-γ ELISPOT in patients recovering from mild COVID-19 [65,66].

(iv) B-cell mediated immunity. The rapid detection of virus-specific antibodies of IgM, IgG and IgA types in the days following SARS-CoV-2 infection indicates the generation of a robust B cell response in COVID-19 patients [55]. The N and S proteins are the most immunogenic molecules, and therefore, most of the antibodies are directed to these antigens [67,68]. Moreover, the neutralizing antibodies directed to the RBD of the S protein block virus interactions with the entry receptor ACE2 protecting against infection [68]. High SARS-CoV-2-specific antibody titers were shown to inversely correlate with viral loads in COVID-19 patients and to directly correlate with an enhanced in vitro virus neutralization [69,70]. In addition, the B cell population is reduced in patients suffering severe forms of COVID-19 [7,25,56,57]. In children, virus specific IgM switched to IgG within 1 week suggesting that this efficient humoral immune response is responsible of the milder symptoms observed in children [71].

All in all, these data indicate that the generation of efficient specific adaptive immune responses together with the development of immunological memory can be a key tool to prevent SARS-CoV-2 infections or reduce their severity. Therefore, COVID-19 vaccines are being developed in a world race as never seen before for any other disease.

### 3.2. SARS-CoV-2 Vaccines

The urgent need for safe and efficacious vaccines to counter the COVID-19 pandemic has accelerated the study, characterization and development of a number of vaccine candidates. Vaccine manufacturers and academic-scientific institutions collaborated globally to develop vaccines against SARS-CoV-2 by using novel and established platforms [72,73]. Some of these experimental vaccines have already progressed into or finished the phase III clinical testing [4]. In broad terms, two components are needed in order to develop efficient vaccines: an antigen delivery system that is capable to deliver the antigens of the target pathogen to the body site in which the immune response will be generated initially and an adjuvant which provides the signal(s) to activate the host immune system [74]. The different vaccine platforms used for the development of vaccines against COVID-19 utilize different strategies as antigen delivery vectors and adjuvants. These platform technologies include the inactivation or attenuation of live SARS-CoV-2, recombinant antigens or synthetic peptides, nucleic acid based (DNA and RNA) vaccines and non-replicating and replicating viral vectors [4,5].

High titers of the infectious virus are needed for conventional inactivated vaccines. Then, this strategy requires the massive cultivation of SARS-CoV-2 in facilities with a biosafety level 3, which have major safety and logistic concerns. Of note, the incomplete inactivation of SARS-CoV-2 poses a risk for vaccine production workers and vaccinated people, particularly those in the high-risk populations such as the elderly and patients with comorbidities [4,5]. In addition, the process of virus inactivation may induce modifications in antigenic epitopes, making them different from those of the viable virus. To avoid these disadvantages, live-attenuated vaccines can be used as they are capable of inducing immune responses against different antigens (real epitopes) of the pathogen. A drawback of this kind of vaccines is the risk of recombination of the live attenuated virus with a wild-type coronavirus [4,5]. Several of COVID-19 vaccine candidates in preclinical testing or in use are based on the platforms of non-replicating adenoviral vectors [4], which have shown high efficacies. In spite of this, this kind of vaccine presents some limitations, such as the development of immune responses against the viral vector restricting boosting or future applications of the same virus. Moreover, the pre-existing immunity against the viral vector can render a vaccine ineffective [5].

All these concerns can be avoided by using subunit vaccines. In this approach, vaccines are formulated with defined recombinant proteins or synthetic peptides. Since subunit vaccines include specific viral antigenic fragments and do not contain other pathogenic components, they are generally considered highly safe [4,5,75]. Due to the low immunogenicity of subunit vaccines, the use of potent adjuvants is mandatory. Moreover, the adjuvant incorporated in the vaccine formulation should be carefully selected, since the immune responses generated with this kind of vaccine are heavily dependent on the adjuvant used. As mentioned before, the RBD of the S protein of SARS-CoV-2 plays a vital role in the infection of the target cells. Thus, antibodies directed to the S protein or RBD could efficiently protect against infection [75]. In line with this, it was previously reported that the antibodies directed to the S protein of SARS-CoV are able to neutralize the virus and prevent infection [76]. On the other hand, almost all SARS-CoV-2-infected persons produce IgM, IgG and IgA antibodies against the S protein between 1–2 weeks after the first symptoms [5]. It was suggested that neutralizing antibodies induced by SARS-CoV-2 infection, especially those directed to the epitopes present in the S protein, plays a crucial role in controlling viral infection and preventing reinfections [77] and are the principle of protection, resulting from convalescent plasma treatments [78]. Hence, the S protein constitutes a major target antigen for SARS-CoV-2 subunit vaccine candidates [5,75].

The S protein is composed of the S1 and S2 domains (Figure 1). The S1 domain is membrane distal and contains the RBD that binds to the host receptor ACE2 [3]. The S protein in different forms, including full-length S protein, S1-RBD or RBD, generates antibody responses in animal models and non-human primates and were shown to confer protection against SARS-CoV-2 infection [75]. While antibodies directed to S1-RBD or RBD block the interaction of the virus with ACE2, antibodies that target other regions of the S1 or S2 domains can inhibit the conformational changes of the S protein and block membrane fusion [75].

The evaluation of the anti-S protein IgG antibodies titers is highly variable among COVID-19 cases, ranging from undetectable to values superior to 100,000 [5]. The pathological consequences of the infection, which could limit the development of appropriate antibody responses, were suggested as a cause of the wide variation in antibody titers seen in COVID-19 patients. In addition, strong antibody responses do not necessarily correlate with mild forms of the disease. Some clinical studies reported the highest antibody titers in the patients who later developed the most severe cases of COVID-19 requiring hospitalization [5]. In contrast, people with mildly symptomatic infection generally had weaker antibody responses. High levels of neutralizing antibodies have been observed in convalescent individuals [65], which correlate with CD4^+^ T cell responses [79]. Of the patients recovered from COVID-19, 100% had S protein-specific CD4^+^ T cells and 70% had S protein-specific CD8^+^ T cells in blood samples [79]. Moreover, some studies reported that persons recovered from COVID-19 seem to have high levels of both neutralizing antibodies and T cells, and compared with severe cases, milder cases of COVID-19 have greater numbers of memory CD8^+^ T cells in the respiratory tract [59,65,79]. This evidence suggests that both antibody-mediated and T cell-mediated immunity are required for the effective protection against SARS-CoV-2 [3].

DNA and RNA vaccines contain selected genes of the SARS-CoV-2, and following cytosolic delivery, these genes are translated into viral proteins that induce a protective immune response. The nucleic acids-based vaccines have relevant advantages regarding producibility, stability and storage. Although these types of vaccines have been proved to induce both humoral and cellular immunity, the general experience and the combined emerging data of SARS-CoV-2 suggests that they may be the least capable of eliciting high titers of antibodies in comparison with the other vaccine platforms [5]. In addition, there is no clear evidence yet whether DNA or RNA vaccines would be capable of eliciting mucosal protective immunity [4,5].

All the previously mentioned vaccines are valuable tools to combat COVID-19. Still, there are no reports on the generation of mucosal immunity. Thus, there still an open path for a new generation of vaccines with the ability to impact the mucosal immune system, to avoid the mucosal replication of the virus and its dissemination.

## 4. *L. plantarum* as Modulators of Antiviral Immune Responses in Mucosal Tissues

The intestinal microbiota plays a key role in maintaining mucosal antiviral immunity in both local mucosal tissues (intestinal mucosa) and in distal mucosal sites (respiratory mucosa) [80,81,82,83]. Of note, most scientific works highlighted the ability of the intestinal microbiota to modulate the innate antiviral defense mechanisms of the gut and the respiratory tract immune by interacting with epithelial and antigen presenting cells. Furthermore, this modulation of the mucosal innate immune response also influences the mucosal antiviral cellular and humoral adaptive immune responses [82,84,85]. Remarkably, not all the members of the intestinal microbiota contribute equally to the beneficial modulation of the mucosal antiviral immunity. This opened the possibility of exploring particular strains of beneficial bacteria with immunomodulatory capacities, referred to as immunobiotics, in order to increase antiviral defenses in mucosal tissues [82,84,85]. Among these beneficial microorganisms with immunomodulatory capabilities, some *L. plantarum* strains are interesting alternatives to improve local and distal antiviral immune responses when orally administered [86,87,88].

### 4.1. Modulation of Intestinal Antiviral Immune Responses by L. plantarum

**Figure 3:**
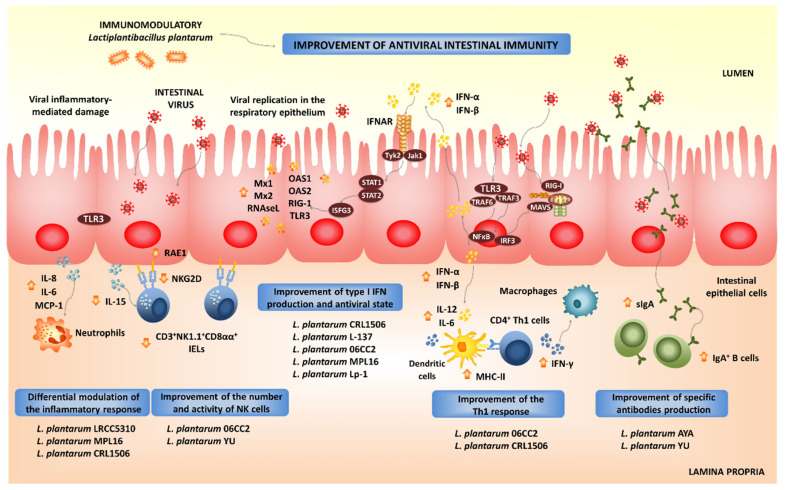
Beneficial effects of orally administered immunobiotic *Lactiplantibacillus plantarum* strains on the resistance and immune responses against virus in the intestinal mucosa.

The interactions of epithelial cells with microorganisms reaching the intestinal mucosa play a central role in determining the type of immune responses that those microbes will trigger [89,90]. For viruses, the early intestinal innate response is the secretion of type I IFNs and the production of several ISGs that exert antiviral activities. Intestinal beneficial microorganisms have been shown to improve these innate antiviral mechanisms [82], and therefore, the microbe-intestinal epithelial cell interaction has been used as an effective tool for selecting immunobiotics to help fight intestinal viral infections. In this regard, we demonstrated that an originally established porcine intestinal epithelial cell line (PIE cells) is able to respond to ligands of the TLR3 and RIG-I receptors as well as to rotavirus infection [91,92]. Furthermore, the in vitro PIE cells system serves for screening of immunobiotic LAB strains with the capacity to differentially modulate IFN-β production and ISGs expression upon poly(I:C) stimulation or rotavirus challenge [82]. Our studies in PIE cells demonstrated that the *L. plantarum* strains CRL1506 and MPL16 increased IFN-γ and IFN-β levels with a concomitant up-regulation of the ISGs, while other *L. plantarum* strains such as CRL681 could not [87,88,93]. Among the ISGs up-regulated by *L. plantarum* CRL1506 and MPL16, a notable effect was observed for Mx2 and RNAseL, which are important for the protection of the intestinal mucosa against viruses [94]. These and probably several other ISGs induced by CRL1506 and MPL16 strains through the IFN-α/β pathway would be related to the lower rotavirus replication found in lactobacilli-treated PIE cells. We confirmed in vivo the antiviral immunomodulatory properties of *L. plantarum* CRL1506 and MPL16 by using mice models [87,95,96]. Both strains significantly increased the production of intestinal type I IFNs and IFN-γ in mice after the challenge with the TLR3 agonist poly(I:C). In line with this, other studies reported the capacity of *L. plantarum* strains to enhance the antiviral immunity in the intestinal epithelium (Figure 3). The antiviral immunomodulatory properties of *L. plantarum* Lp-1 was studied in pig jejunal cells (IPEC-J2 cells) infected with the coronavirus transmissible gastroenteritis virus (TGEV), which is one of the most important gastrointestinal pathogens causing diarrhea, vomiting and high mortality in piglets [97]. The experimental data showed that the TGEV titers in IPEC-J2 cells treated with *L. plantarum* Lp-1 were significantly lower than controls. This effect was related to the ability of the Lp-1 strain to augment the expression of IFN-β, ZAP, MX2, MX1, PKR, OASL and ISG15 in IPEC-J2 cells after TGEV infection [97].

The activation of PRRs-mediated signaling pathways in intestinal epithelial cells not only stimulates the production of type I IFNs and ISGs, but also the expression of pro-inflammatory cytokines and chemokines. The activation of the NF-κB signaling pathway stimulates the production of inflammatory cytokines, including IL-1β, IL-8, GM-CSF, CCL-3 and CXCL-10 in the intestinal epithelium after viral infection [98,99]. This inflammatory response is the first line of host defense against viruses: it recruits and activates immune cells; however, if it is deregulated or it extends excessively in time, it may lead to tissue damage and epithelial barrier dysfunction [98,99]. Then, the efficient regulation of intestinal inflammatory responses induced by virus is essential to achieve full protection against the infections. Immunobiotics can also help in the modulation of intestinal inflammatory responses. We demonstrated that *L. plantarum* strains CRL1506 and MPL16 differentially modulated the production of pro-inflammatory mediators in PIE cells upon the activation of TLR3 [87,88,93]. Moreover, both CRL1506 and MPL16 strains reduced TLR3-induced small intestinal injury in mice by regulating the production of pro-inflammatory cytokines and the interaction of intestinal epithelial cells and intraepithelial lymphocytes [87,95]. It was shown that the abnormal TLR3 signaling induce the expression of IL-15 in the intestinal epithelium, leading to the activation of CD3^+^NK1.1^+^CD8αα^+^ intraepithelial lymphocytes, which increase the apoptosis of intestinal epithelial cells induced by the perforin pathway [100]. In mice, the administration of poly(I:C) activates TLR3 in the intestinal mucosa, inducing villous atrophy and mucosal erosion [101]. In line with these findings, it was demonstrated that the blocking of the IL-15 receptor partially protected mice from poly(I:C)-induced small intestinal injury [101]. The activation of TLR3 stimulates intestinal epithelial cells to express retinoic acid early inducible 1 (RAE1) and induces CD3^+^NK1.1^+^CD8αα^+^ cells to express NKG2D through IL-15 [102]. Then, the RAE1-NKG2D interaction has a prominent role in the intestinal injury. Interestingly, we demonstrated that mice pretreated with *L. plantarum* CRL1506 before TLR3 activation responded with reduced levels of IL-15 and RAE1 in intestinal epithelial cells and with diminished expression of NKG2D in CD3^+^NK1.1^+^CD8αα^+^ intraepithelial lymphocytes [95]. Moreover, mice treated with CRL1506 or MPL16 strains before a poly(I:C) challenge significantly reduced the levels of intestinal TNF-α, IL-1β and IL-8 [87,95]. The beneficial effect of the *L. plantarum* strains in the intestinal inflammatory injury were translated into significant reductions of body weight loss and intestinal histological damage after poly(I:C) challenge.

*L. plantarum* LRCC5310, and particularly its exopolysaccharides (EPSs), induces an antagonistic effect against human rotavirus. MA104 cells treated with the LRCC5310 strain or its EPSs had significantly lower cytopathic alterations and reduced viral replication when compared to untreated controls [103]. In addition, orally administered *L. plantarum* LRCC5310 increased the protection of young mice against rotavirus infection reducing the duration of diarrhea and viral shedding and preventing the destruction of the intestinal epithelium integrity [103]. Complementary in vitro studies demonstrated that *L. plantarum* LRCC5310 EPS increases IL-10 and reduces IL-1β and TNF-α in both intestinal epithelial cells and macrophages [103]. A clinical trial where *L. plantarum* LRCC5310 was administered in infants with rotavirus enteritis did not show significant differences between control and LRCC5310-treated groups at the beginning of the study in terms of clinical symptoms and laboratory inflammation markers. However, at the end of the trial, there was a significant reduction in the severity and duration of diarrhea as well as rotavirus titers in LRCC5310-treated children when compared to controls [104].

We also demonstrated that *L. plantarum* CRL1506 modulated the expression of pro-inflammatory cytokines and influenced the activation and maturation of intestinal antigen presenting cells [93]. It has been reported that DCs produce IFN-α/β and undergo maturation in response to type I IFNs. Moreover, IFN-α/β have been shown to potently enhance the activation of DCs in vivo, serving as an important link between innate and adaptive immunity in the context of viral infections [105]. In fact, the in vitro treatment of DCs with type I IFNs activates these cells and increases their capacity to initiate T cell responses. DCs stimulated with type I IFNs have improved expression of MHC-II as well as the co-stimulatory molecules CD40 and CD86. Furthermore, IFN-treated DCs had a higher ability to stimulate CD4^+^ and CD8^+^ T cells that produce IFN-γ [106,107]. In line with these reports, our in vitro and in vivo results demonstrated the immunobiotic *L. plantarum* strains CRL1506 and MPL16 were able to improve intestinal Th1 response, as evidenced by the augmented expression of MHC-II, IL-1β, IL-6 and IFN-γ in the intestinal DCs [87,88,93]. Thus, *L. plantarum* would be capable of stimulating the intestinal adaptive immunity through its ability to modulate antigen presentation in DCs in a type I IFN-dependent manner. Moreover, this hypothesis prompted us to evaluate whether orally administered *L. plantarum* CRL1506 or its non-viable bacterium-like particles (BLPs) were able to modify the immune response to an oral vaccine [108]. In our hands, *L. plantarum* CRL1506 or its BLPs showed adjuvant capacities when used together with a rotavirus vaccine. Immunization of mice with *L. plantarum* CRL1506 or its BLPs significantly improved the specific Th1 mucosal and systemic immune responses generated against the rotavirus antigens. Furthermore, our immunization protocol not only stimulated the cellular immunity but also increased levels of intestinal IgA- and serum IgG-specific antibodies were found in animals immunized with rotavirus vaccine and the CRL1506 strain [108]. These results provide additional evidence for the hypothesis that gives a key role to type I IFNs in the efficient generation of adaptive responses induced by immunobiotic *L. plantarum* treatments since it was demonstrated that type I IFNs promote antibody responses in vivo [109]. In fact, type I IFNs were shown to increase the synthesis of antigen-specific antibodies of all subclasses of IgG and induced IgG2a and IgG3 antibodies far more effectively than widely used adjuvants [109]. In line with our results, the oral administration of *L. plantarum* 06CC2 significantly increased IFN-γ and IL-12p40 expression in Peyer’s patches of unchallenged [110] as well as in Herpes Simplex Virus type 1 (HSV-1)-infected mice [111]. Furthermore, the lactobacilli treatment improved splenic NK cell activity and IFN-γ-producing cells. This immunomodulatory effect induced by the 06CC2 strain correlated with reduced HSV-1 titers in skin lesions.

Of note, our and other groups proved that *L. plantarum* strains had different adjuvant capacities in terms of their ability to improve the intestinal cellular and humoral immune responses triggered by virus infection or vaccination. Both humoral and cellular intestinal specific immune responses were significantly upgraded in mice immunized with rotavirus vaccine and *L. plantarum* CRL1506 but not in animals treated with *L. plantarum* CRL1905 or its BLPs, indicating that distinct strains function differently as mucosal adjuvants [108]. The final outcome of cellular response against beneficial immunomodulatory lactobacilli depends on the combination of different microbial-associated molecular patterns (MAMPs) that can interact with various PRRs to trigger different signaling pathways [112]. The unique combination of cellular and molecular interactions established between certain lactobacilli strains with non-immune and immune cells explains why the immunomodulatory properties of LAB in general, and *L. plantarum* in particular, are a strain dependent characteristic. Then, an efficient selection of the most effective *L. plantarum* strains with the ability to beneficially modulate intestinal immunity is necessary, in order to use them in the development of functional foods or as adjuvants for mucosal vaccines. This becomes more relevant if the desired functional food or vaccine formulation aims to confer immunity not only in the intestinal mucosa but also in distant mucosal sites such as the respiratory tract, since this effect is only achieved by a small group of strains, as outlined below.

### 4.2. Modulation of Respiratory Antiviral Immune Responses by L. plantarum

**Figure 4:**
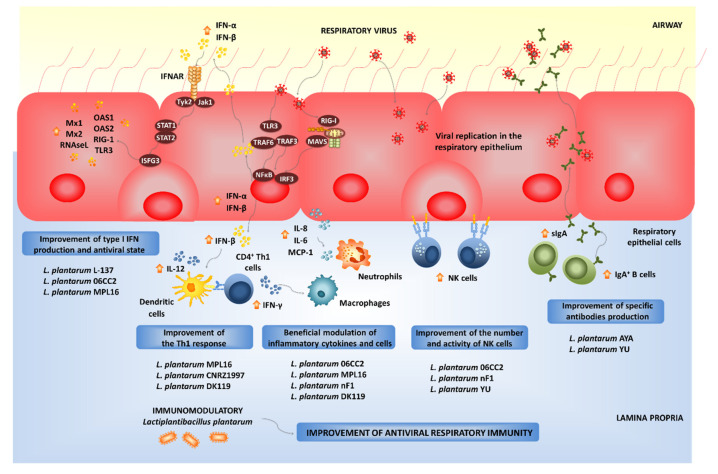
Beneficial effects of orally administered immunobiotic *Lactiplantibacillus plantarum* strains on the resistance and immune responses against viruses in the respiratory mucosa.

Experiments in animal models and human clinical trials have demonstrated that certain LAB strains, when orally administered, are effective in modulating the respiratory immunity and enhancing the resistance against bacterial and viral infections. This group of immunobiotic LAB improve the resistance of children, adults and the elderly to respiratory infections such as the pneumococcal pneumonia-, the common cold- and influenza-like symptoms [85]. Among the LAB strains that possess this unique immunomodulatory property, there is a group of strains belonging to the species *L. plantarum* (Figure 4).

Not all lactobacilli strains possessing immunomodulatory abilities in the intestinal mucosa are capable of stimulating the respiratory immunity when orally administered [110,111,112,113,114,115,116]. Orally administered *L. plantarum* MPL16 but not *L. plantarum* CRL1506 was capable of modulating respiratory immunity. The MPL16 strain is capable of improving the levels of IFN-γ and type I IFNs in the respiratory tract, indicating its ability to modulate the function of CD4^+^IFN-γ^+^ T cells and CD11c^+^SiglecF^+^ alveolar macrophages. Moreover, the increased levels of respiratory IFN-γ and IFN-β found in *L. plantarum* MPL16-treated mice correlated with the improved resistance of mice to RSV infection [87].

The strain-dependent ability of orally administered LAB to modulate the respiratory immunity was also confirmed by other research groups. The capacity of several *L. plantarum* strains to modulate the respiratory immunity when orally administered was evaluated in a lethal model of IFV pneumonia [110]. Among the strains evaluated, *L. plantarum* 06CC2 stood out for its ability to improve the survival of mice infected with IFV, while the *L. plantarum* strains 05AM23, 06TCa8, 06TCa40 and 06CC9 induced no protective effect. Orally administered 06CC2 strain reduced IFV titers in lungs and improved Th1 response in the respiratory tract and NK cell activity in both lungs and spleens. In addition, in a large-scale screening of immunomodulatory LAB, several *Lactiplantibacillus* strains were evaluated in their ability to modulate immunity in both TNF-α-activated HT-29 cells and peripheral blood mononuclear cells [116]. Among the evaluated strains, *L. plantarum* CNRZ1997 was able to significantly increase the production of inflammatory cytokines in both epithelial and immune cells, while other *L. plantarum* strains showed no effect or demonstrated to have an anti-inflammatory capacity. Interestingly, the work demonstrated that the orally administered CNRZ1997 strain was capable of reducing the replication of IFV in the respiratory tract of mice. The oral treatment with *L. plantarum* CNRZ1997 was as effective as the oral treatments with *L. rhamnosus* GG or *L. casei* DN114-001 (two commercial probiotic strains with anti-IFV properties in preclinical and human trials) to protect mice against IFV infection [84].

It seems that viability is not a requisite for the beneficial modulation of the antiviral respiratory immunity, as was seen with heat-killed *L. plantarum* L-137, which improved Th1 immunity in healthy subjects [117]. The strain was capable of increasing concanavalin A-induced proliferation of blood lymphocytes and enhancing the percentages of CD4^+^IFN-γ^+^ T cells. Orally administered heat-killed *L. plantarum* L-137 beneficially modulated antiviral immunity in mice [118] and pigs [119], and stimulated the production of IFN-β in mice following IFV infection [118]. Further studies in pigs demonstrated that the L-137 strain significantly improved the expression of IFN-β in blood cells [119]. The treatment with *L. plantarum* L-137 was translated into a higher body weight gain of pigs when compared to controls, indicating an improved health status. The impact of the L-137 strain on upper respiratory tract infections was evaluated in human subjects with high psychological stress levels [120]. *L. plantarum* L-137 reduced the incidence, duration and severity of respiratory infections as well as the duration of medication. Similarly, the consumption of *L. plantarum* nF1-fortified yogurt improved IL-12 and IFN-γ production and NK cell activity in elderly subjects, indicating its potential to stimulate antiviral immunity [121]. In fact, the oral treatment of mice with heat-treated *L. plantarum* nF1 significantly increased the expression of IFN-γ, IL-2 and IL-12 in the spleen, the NK cell membrane marker Klrb1 and the NK and T cells activation marker CD69 after the challenge with IFV [122]. In line with these findings, a higher splenic NK activity was observed in nF1-treated mice. Those immunological changes induced by *L. plantarum* nF1 correlated with a reduction of IFV titers and an improved survival of mice [122,123].

Alveolar macrophages are key cells in the beneficial modulation of respiratory immunity induced by gut microorganisms. It was shown that the intestinal microbiota help to maintain the optimal antiviral functions of alveolar macrophages. Beneficial gut microbes are involved in the efficient capacity of alveolar macrophages to produce type I IFNs and antiviral factors including IRF7, IFNGR1, STAT1, STAT2, IFIT3, MX1 and OAS1 that improve the resistance to respiratory viral infections [81]. We have recently demonstrated that immunobiotics with the ability of modulating the respiratory immunity are able to functionally modulate the alveolar macrophages response to viral challenges. In fact, our results indicate that alveolar macrophages greatly contribute to the augment of IFN-γ and IFN-β in the respiratory tract of mice orally treated with *L. rhamnosus* CRL1505 [115] or *L. plantarum* MPL16 (submitted for publication). Moreover, an improved expression of IFNAR1, Mx2, OAS1, OAS2, RNAseL and IFITM3 in alveolar macrophages after the oral treatment with *L. rhamnosus* CRL1505 or *L. plantarum* MPL16 was detected in our experiments. In line with our results, it was reported that mice orally treated with *L. plantarum* DK119 had higher BAL IL-2 and IFN-γ levels, and a low degree of inflammation upon IFV infection [124]. The lactobacilli treatment reduced viral loads in the lungs and improved survival of infected mice. In contrast, the levels of IL-6 and TNF-α in the respiratory tract of DK119-treated mice were lower compared to those from control mice after IFV infection. Consistent with this pattern of cytokines, a significantly reduced degree of inflammation was observed in mice receiving *L. plantarum* DK119. In mice treated with clodronate liposomes to induce the depletion of CD3^-^CD11b^-^CD11c^+^F4/80^+^ alveolar macrophages, the administration of the DK119 strain could not induce modifications in the severe weight loss and mortality induced by IFV infection, indicating that alveolar macrophages have a key role in *L. plantarum* DK119-mediated protection [124].

The exact nature of the cellular and molecular signals used by orally administered immunobiotics to modulate the respiratory antiviral immunity remain to be determined. Our recent experiments blocking CD4^+^ T cells and IFN-γ allow the speculation of a potential mechanism that could explain the remote effect induced by orally administered immunobiotics [115]. The existence of the so-called common mucosal immune system implies that the immune cells activated in one mucosal tissue can mobilize and reach distant mucosal sites, where they can influence immune responses. Then, the mobilization of B and T cells from the intestinal mucosa to the respiratory tract could be involved in the beneficial effects exerted by orally administered immunobiotics [85,115]. Strains such as *L. plantarum* MPL16 would induce the mobilization CD4^+^IFN-γ^+^ T cells from the intestine to the lungs, and the local production of IFN-γ would modulate the respiratory tract innate immune microenvironment, leading to the activation of local immune cells such as alveolar macrophages. As mentioned in the previous section, *L. plantarum* 06CC2 enhances the expression of IFN-γ and IL-12 in Peyer’s patches, as well as in the respiratory tract supporting the hypothesis that the 06CC2 strain elicited its protective effect in the respiratory tract through intestinal immunity [110]. On the other hand, *L. plantarum* YU was shown to strongly induce the in vitro production of IL-12 by antigen presenting cells from murine Peyer’s patches, mesenteric lymph nodes and spleen [125], which was associated to TLR2 stimulation. Orally administered *L. plantarum* YU was able to reduce body weight loss and lung viral replication after a challenge with IFV, to enhance intestinal IgA concentration and splenic NK cell activity. Of note, the levels of IFV-specific secretory IgA in BAL samples were also increased by the oral treatment with *L. plantarum* YU [125]. Several LAB strains were evaluated according to their ability to stimulate the production of IgA in primary cultures of immune cells isolated from mice Peyer’s patches [126]. Among the strains evaluated, *L. plantarum* AYA and N63 had the ability to significantly increase IgA levels in vitro. However, when the two strains were evaluated in vivo, only *L. plantarum* AYA increased the levels of intestinal IgA in orally treated mice. Of note, the immunomodulatory effect was obtained with both the viable bacterium as well as the heat-killed *L. plantarum* AYA. The oral administration of the AYA strain stimulated DCs by modulating their expression of IL-6 and significantly increased the numbers of IgA^+^B220^+^ cells in mice Peyer´s patches. Mice treated with AYA strain and then challenged with IFV had an improved resistance to the viral infection. This protective effect was associated to the induction of higher levels of IgA in the respiratory tract [126]. The work suggested that *L. plantarum* AYA stimulate IL-6 production in intestinal DCs, which in turn promotes the differentiation of IgA^+^ B cells into plasma cells improving intestinal IgA production (Figure 3). Moreover, *L. plantarum* AYA would also induce the mobilization of IgA^+^ B cells from the intestinal mucosa to the respiratory tract, enhancing the IgA production in the context of a viral respiratory infection (Figure 4).

All in all, these data indicate that appropriate immunobiotic *L. plantarum* strains could be used to improve antiviral immunity on both the intestinal and the respiratory mucosa at the same time, and therefore, they are an interesting biotechnological resource for the development of mucosal vaccines.

## 5. *L. plantarum* as Platforms for Mucosal Vaccines Development

Currently, there are few approved mucosal vaccines. Most of them constitute attenuated pathogens that need a cold chain and carry the risk of reversion to virulence. Importantly, these mucosal vaccines have limited efficacy in individuals with poor mucosal health [127]. On account of these limitations, new types of mucosal vaccine vectors are necessary, such as recombinant LAB as next-generation mucosal vaccine vectors, due to their natural acid and bile resistance, stability at room temperature and their ability to beneficially modulate mucosal innate and adaptive immune responses. Moreover, the advances in the molecular biology techniques that allow the manipulation of LAB gene expression have allowed scientists to express in them a great variety of molecules with potential application in medicine including pathogens antigens. As vaccine vectors, LAB offer several advantages including simple, non-invasive mucosal administration, low cost and high safety levels. LAB tend to elicit minimal immune responses against themselves, instead inducing high levels of systemic and mucosal antibodies against the expressed foreign antigen following uptake via the mucosal immune system [128]. Furthermore, as highlighted in the previous sections, some orally administered LAB could be used to induce specific immunity not only in the intestinal mucosa but also in the respiratory tract.

LAB for use as vaccine vectors generally include *Streptococcus gordonii*, *Lactococcus lactis* and multiple *Lactobacillus* species. Although most of them are *L. lactis*-based vaccines, the immunogenicity of *L. plantarum*-based vaccines was reported to be significantly higher than that of *L. lactis* [129]. Since recombinant *L. plantarum* expressing heterologous antigens can induce both specific humoral and cellular immune responses against pathogens and toxin in mucosal tissues [129,130,131,132,133,134,135,136] and the oral administration of *L. plantarum* can effectively stimulate antiviral immune responses [87,88], during past decades, *L. plantarum* have been evaluated as vehicles for delivering recombinant viral antigens [131,137,138].

To date, several delivery systems based on *L. plantarum* were established for cell-surface expression of viral antigens (Table 1) [137,138,139]. A cell wall anchoring system for E7 mutant protein (E7mm) of human papillomavirus (HPV) type-16 was constructed by fusing the signal peptide and the first 15 amino acids of lactococcal Usp45 protein at the N terminal of the viral protein, and the cell wall anchor of *L. plantarum* lp_2940 protein at the C terminal [139]. The E7mm protein can be efficiently anchored and displayed on the cell wall of *L. plantarum* via anchor motif LPQTXE [139]. However, the display efficiency of heterologous protein on the cell surface may be affected by signal peptide, surface anchor and host cell surface structure [137,139,140]. As for *L. plantarum*, the heterologous antigen can be displayed on the surface of the bacteria via C-terminal or N-terminal anchors. The C-terminal anchors include sortase-mediated covalent cell wall anchors containing LPXTG or LPQTXE domains, which can be catalyzed by sortase and covalently attached to peptidoglycan on the cell wall [140]. The N-terminal anchors contain lipobox-based covalent cell membrane anchor derived from *L. plantarum*, lysin motif (LysM)-based non-covalent cell wall anchor derived from *L. plantarum*, N-terminal signal peptide-based transmembrane anchor derived from *L. plantarum* and anchoring poly-γ-glutamate synthase A (pgsA) from *Bacillus subtilis* [140,141,142].

The porcine IFN-λ3 was linked with C-terminal cell wall anchor and N-terminal transmembrane anchor pgsA, respectively, and it was found that both strategies anchored the porcine IFN-λ3 on the bacteria surface and stimulate strong antiviral effects against the porcine epidemic diarrhea virus (PEDV) and TGEV in IPEC-J2 cells. However, recombinant *L. plantarum* with the C-terminal cell wall anchor exhibited a more powerful antiviral effect than that of the recombinant *L. plantarum* with pgsA [143].

The cell-surface display efficiency of three surface anchors in eight different *Lactobacillus* were evaluated and it was found that LPXTG- and LysM-based anchors of *L. plantarum* exhibited higher efficiency than that of the lipoprotein anchor [137]. Notably, heterogenicity was observed in antigen display cells and cell populations. Furthermore, the comparative analysis of the surface-display of eight *Lactobacillus* species found that *L. plantarum* and *L. brevis* were the most promising vehicles, which could elicit significant antigen-specific mucosal IgA, IFN-γ and IL-17 [131]. In addition, we previously found that codon optimization of expression cassette can promote the expression of the heterogeneous protein in *L. plantarum* [144]. These results indicate that the surface display needs to be optimized for different antigens and hosts, and homologous anchors from hosts seem to be promising candidates for displaying heterologous proteins.

The strategy of targeting DCs and M cells have been shown to be effective in enhancing mucosal and adaptive immune responses. The specific deliver of heterogeneous antigens expressed in *L. plantarum* to DCs and M cells have been explored recently [132,133,135,144,145,146,147]. Wang and colleagues constructed a series of recombinant *L. plantarum* for delivering specific antigens against the Newcastle Disease Virus (NDV) [132], TGEV [133], PEDV [135], and avian influenza virus H9N2 [134] by fusing the viral antigen with DC-cell target peptide (DCpep), respectively. The results showed that the DCpep-fused antigens significantly increased the production of intestinal IgA, and stimulated proliferation and differentiation of B and T cells by directly targeting the antigen to DC cells. Furthermore, this strategy leads to a significant enhancement in cellular and humoral immune responses [132,133,134,135,144]. In addition, we also found that the porcine reproductive and respiratory syndrome virus (PRRSV) GP5 protein fused with M-cell target peptide (Mpep) and expressed in *L. plantarum* showed slightly higher antigenicity than the DCpep-fused GP5 protein [144].

**Figure 5:**
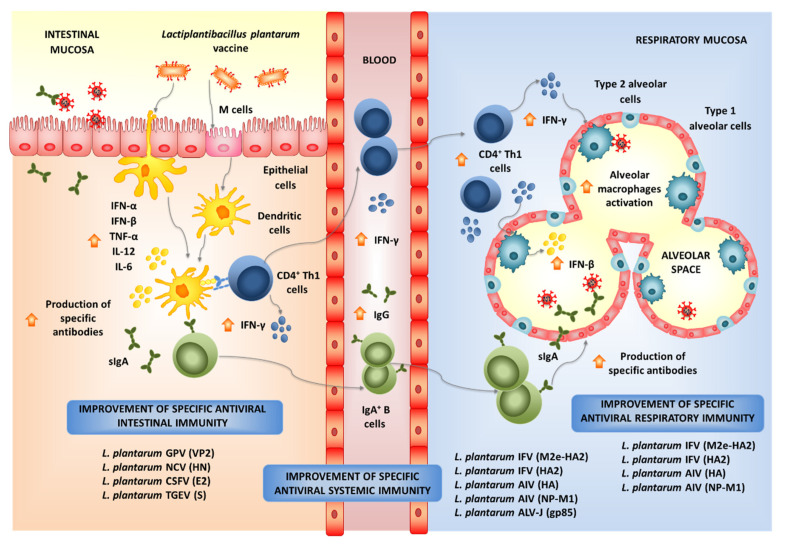
Beneficial effects of orally administered recombinant *Lactiplantibacillus plantarum* strains on the resistance and immune responses against virus in the intestinal and respiratory tissues.

Notably, experimental *L. plantarum*-based vaccines have been shown to induce local as well as distal specific immunity when orally administered (Figure 5). The immunogenicity of a recombinant *L. plantarum* expressing the goose parvovirus (GPV) VP2 antigen was evaluated in BALB/c mice [148]. Mice were orally immunized with the recombinant lactobacilli had a remarkable production of specific IgA in the intestinal mucosa. In addition, the work also described the induction of specific cellular immunity, as demonstrated by the increased levels of TNF-α and IFN-γ by the in vitro stimulation of splenocytes with the recombinant VP2-GPV protein when compared to controls. Chickens orally immunized with the recombinant *L. plantarum* expressing the HN-DCpep of NDV produced both serum anti-HI antibodies and specific intestinal IgA, and had significantly higher proliferation rates of splenic T cells upon HI challenge when compared to control animals [132]. Of note, the resistance of chickens to NDV infection was improved by the immunization protocol. Similarly, the intragastric immunization of chickens with the recombinant *L. plantarum* strain expressing the gp85 protein of the J Avian Leukosis Virus (ALV-J) induced the production of specific serum IgG and intestinal IgA antibodies [141]. Moreover, this immunization treatment significantly reduced the ALV-J viremia when compared with the control groups. The E2 protein of the classical swine fever virus (CSFV) was expressed in *L. plantarum* and the recombinant bacteria was used to immunize pigs by the oral route [149]. The immunization of animals induced the production of specific serum IgG and intestinal IgA as well as specific cytotoxic responses against CSFV. Furthermore, a virus challenge experiment demonstrated the ability of *L. plantarum* E2-CSFV to improve the protection pigs against viral infection as demonstrated by their higher survival rates and lower severity of the symptoms.

The ability of orally administered recombinant *L. plantarum* to improve immune responses in distal mucosal sites was studied mainly with the viral pathogen IFV (Figure 5). An *L. plantarum* strain expressing the hemagglutinin (HA) of the IFV was constructed and the capacity of the recombinant strain to induce specific immunity and protect against the viral infection were evaluated in BALB/c mice [150]. The oral immunization of mice induced the production of specific serum IgG as well as anti-HA IgA antibodies in both the intestinal mucosa and the respiratory tract. The induction of mucosal and systemic effective B cell responses was further demonstrated by the increased levels of FAS^+^PNA^+^B220^+^ B cells in Payer’s patches, mesenteric lymphoid nodes and spleen. The oral immunization with the recombinant lactobacilli generated specific cellular immune responses, as shown by the enhanced cell proliferation rates and IFN-γ production by T cells in the lymphoid nodes and spleen upon restimulation with the HA antigen. Interestingly, the orally administered *L. plantarum* HA-IFV significantly improved the protection of mice against the intranasal challenge with IFV. Immunized mice had lower weight loss, mortality, respiratory virus titers and lung pathology when compared to controls. Those beneficial effects were later improved by expressing a HA-DCpep in *L. plantarum* [151]. Similarly, a recombinant *L. plantarum* expressing the NP-M1-DCpep of IFV was capable of inducing specific systemic and mucosal immunity and protecting mice against the viral challenge after its oral administration [152]. In order to improve protection against different subtypes of IFV, recombinant lactobacilli expressing the fusion of two viral antigens were developed. Chickens orally immunized with a recombinant *L. plantarum*, expressing the fusion M2e and HA2 antigens from IFV were protected against the pathogen as shown by the decreased pulmonary virus titers and reduced lung and throat pathological damages after the infectious challenge [153]. The oral immunization of mice with *L. plantarum*, expressing the fusion antigen HA2 and 3M2e from IFV, stimulated mucosal and systemic specific immunity, increasing the resistance of mice to the respiratory challenge whit the viral pathogen [154].

These works showed that the oral administration of recombinant *L. plantarum* strains can offer protection against infection by gastrointestinal and respiratory virus by enhancing specific immunity. This strategy is extremely interesting to improve resistance against viruses that can efficiently replicate and infect both mucosal tissues (intestinal and respiratory), such as SARS-CoV-2.

### The Successful Expression of SARS-CoV-2 Antigen in L. plantarum

As mentioned before, more than 180 vaccines are being developed at various stages, including inactivated vaccines, live-virus vaccines, recombinant protein vaccines, DNA or mRNA vaccines and vector-based vaccines [155]. As the S protein of the SARS-CoV-2 is the main antigen with the ability to interact with the host ACE2 initiating viral infection, the S protein has been the main antigenic target for vaccines against the virus [155,156]. As mentioned before, SARS-CoV-2 infection can induce both mucosal and systemic antibody responses, with high specific IgA in upper respiratory tract and intestinal tract of severe- and mild-COVID-19 patients [155,157], suggesting mucosal immune response plays critical role against the virus infection. Therefore, the display and presentation of the viral S protein in upper respiratory tract and the intestinal tract may induce effective mucosal immune response against SARS-CoV-2, which would be capable of reducing the severity of the disease and avoid viral dissemination.

**Figure 6:**
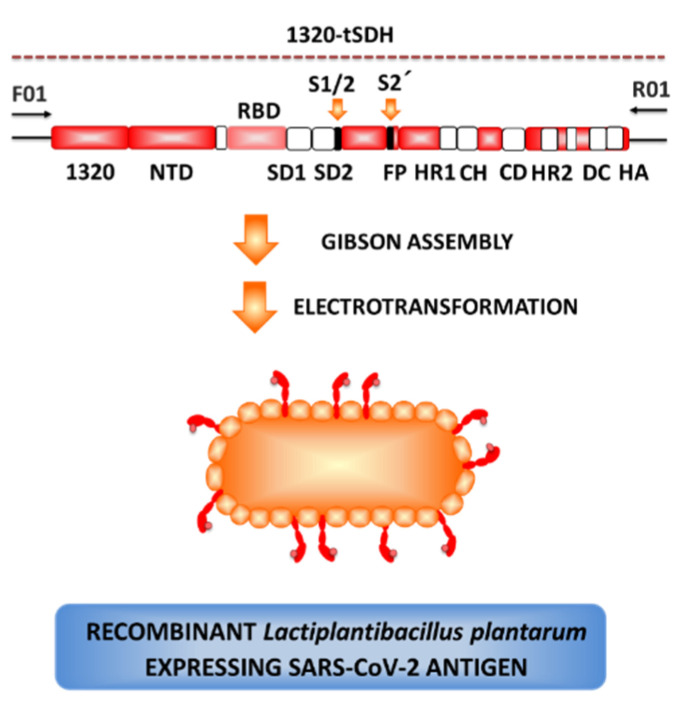
Schematic diagram of the construction of the recombinant *Lactiplantibacillus plantarum* strain expressing the SARS-CoV-2 spike protein. Modified from Wang et al. [161].

We previously demonstrated that *L. plantarum* LP18 (also named CGMCC 1.557) is a promising immunobiotic strain due to its high adhesion to intestinal cells and its remarkable immunoregulatory functions [138,158,159,160]. Then, a recombinant *L. plantarum* LP18, which can display the S protein of SARS-CoV-2 on the bacterial surface, was constructed by fusing sequences of the endogenous signal peptide at the 5′ terminus and the target peptide DCpep at the 3′ terminus of the optimized S gene (Figure 6) [161]. The in vivo evaluation of this experimental vaccine has shown that the antigen displayed by *L. plantarum* can significantly stimulate the mucosal immune response against SARS-CoV-2 infection (unpublished data), further emphasizing that the mucosal-targeted vaccines based on recombinant *L. plantarum* is a promising candidate vaccine for COVID-19. The evaluation of the effectiveness and safety of the recombinant *L. plantarum* is still in progress.

## 6. Conclusions

Generally, systemic vaccination through the subcutaneous, intramuscular or intraperitoneal routes are not capable to induce specific mucosal immunity. Then, the host’s adaptive immune system can only fight the pathogenic microorganisms after they have gained entry into deeper tissues of the body. Thus, the development of vaccines that stimulate both the mucosal and systemic immune systems rather than only inducing a specific systemic immune response would be remarkably advantageous to control pathogens at their point of entry. With this strategy, it would be possible not only to avoid the symptoms associated with infections of the mucosal tissues but also to avoid the spread of the pathogen. These general considerations of systemic vaccines seem to apply to almost all the vaccines in development or in use intended to combat COVID-19.

The induction of mucosal immunity initiates by antigens being taken up by local antigen-presenting cells such as DCs. In the gut, antigens are processed by intestinal DCs, which then migrate to the mesenteric lymph nodes and stimulate the induction of antigen-specific T and B lymphocytes. Upon activation, antigen-specific lymphocytes undergo proliferation and differentiation and exit the mesenteric lymph node via the efferent lymph, and via the thoracic cavity, they can enter into the blood circulation. In this way, the majority of activated antigen-specific T and B lymphocytes come back to the intestinal mucosa. Interestingly, a portion of these activated immune cells is capable to reach mucosal tissues distant from the local intestinal mucosa such as the respiratory tract. The induction of effector and/or memory T and B cells in the intestinal and respiratory tracts by oral immunizations would be tremendously valuable for the protection against pathogens capable of replicating in both mucosal tissues. Although SARS-CoV-2 replicates and affects mainly the respiratory tract mucosa, clinical and experimental works have demonstrated the ability of this virus to infect the gastrointestinal tract. Reports have indicated that a notable proportion of patients with COVID-19 develop gastrointestinal symptoms, while nearly half of patients confirmed to have COVID-19 have shown detectable SARS-CoV-2 RNA in their fecal samples [162]. In addition, multiple in vitro and in vivo animal studies have provided direct evidence of intestinal infection by SARS-CoV-2 [162]. Therefore, while the investigations of the impact of the virus on the intestinal mucosa are progressing, the design of a mucosal vaccine based on immunomodulatory LAB that can induce protective immunity in both the intestinal and respiratory tracts would be of great importance to advance in the fight against SARS-CoV-2.

The hostile environment of the gastrointestinal tract, which includes the stomach extreme pH and the intestinal protease-rich environments, can severely affect the immunogenicity of ingested antigens. In addition, the gastrointestinal immune system has the propensity to respond with tolerance to oral antigens rather than inducing effector immune responses. These characteristics have made the generation of efficient oral vaccines extremely challenging, due to the difficulty of finding appropriate antigen delivery systems and adjuvants that efficiently stimulate mucosal immunity.

As reviewed here, the biotechnological and immunological research of the last decades has demonstrated the potential of *L. plantarum* to be used in the generation of mucosal vaccines. Nowadays, it is possible to express heterologous proteins in LAB, such as the SARS-CoV-2 spike protein in *L. plantarum*. Other recombinant *L. plantarum* strains were proven to induce protective immune responses in the gut when used in oral immunizations, highlighting the capacity of the bacterium to protect the expressed antigens from the hostile gastrointestinal environment. In addition, significant progress has been made in the understanding of the cellular and molecular mechanisms involved in the improvement of mucosal antiviral defenses by beneficial *L. plantarum* strains, which would allow in the near future the improvement of the recombinant lactobacillus-based vaccines design to increase their efficiency. Furthermore, studies have shown the ability of immunobiotic and recombinant *L. plantarum* strains to stimulate the common mucosal immune system allowing the induction of protective immunity not only in the gut but also in the respiratory tract after their oral administration. These scientific advances clearly indicate the potential of *L. plantarum* to be used in the development of a mucosal COVID-19 vaccine, which could be used as a complement to the current systemic vaccines, to ameliorate the symptoms associated with mucosal infections and collaborate in reducing the spread of SARS-CoV-2.

## Figures and Tables

**Table 1:** Summary of viral antigens successfully expressed in *Lactiplantibacillus plantarum*. Human papillomavirus type-16 (HPV-16), J subgroup Avian Leukosis Virus (ALV-J), porcine epidemic diarrhea virus (PEDV), transmissible gastroenteritis virus (TGEV) and Newcastle disease virus (NDV).

Virus	Antigen and Expression Cassette	Location of the Antigen	Route of Administration	Efficacy and Safety of Vaccine	Reference
HPV-16	Signal peptide and the first 15 amino acids of lactococcal Usp45 protein, the E7 mutant protein of HPV-16 E7mm and the cell wall anchor of *L. plantarum* protein containing anchor motif LPQTXE	Cell wall anchoring	-	The effcicacy or the safety of the experimental vaccine based on *L. plantarum* was not reported.	[139]
ALV-J	Anchorin Poly-γ-glutamate synthase A (pgsA) and the ALV-J gp85	Bacterial surface	Oral immunization	Pre-clinical stage. Immunization of chickens stimulated humoral immunity and improved protection against ALV-J challenge. No adverse effects were detected.	[141]
PEDV	Anchorin pgsA, the spike protein of PEDV and target peptide DCpep (FYPSYHSTPQRP)	Bacterial surface	Oral immunization	Pre-clinical stage. Immunization of mice stimulated humoral and cellular immunity. No adverse effects were detected.	[135]
TGEV	Anchorin pgsA, the spike protein of TGEV and target peptide DCpep (FYPSYHSTPQRP)	Bacterial surface	Oral immunization	Pre-clinical stage. Immunization of mice stimulated humoral and cellular immunity. No adverse effects were detected.	[133]
NDV	Hemagglutinin–neuraminidase protein and target peptide DCpep	Intracellular	Oral immunization	Pre-clinical stage. Immunization of chickens stimulated humoral and cellular immunity and improved protection agains NDV challenge. No adverse effects were detected.	[132]

## Data Availability

Not applicable.
